# Prolonged non-survival in PICU: does a do-not-attempt-resuscitation order matter

**DOI:** 10.1186/1471-2253-13-43

**Published:** 2013-11-17

**Authors:** Kam Lun E Hon, Terence Chuen Wai Poon, William Wong, Kin Kit Law, Hiu Wing Mok, Ka Wing Tam, Wai Kin Wong, Hiu Fung Wu, Ka Fai To, Kam Lau Cheung, Hon Ming Cheung, Ting Fan Leung, Chi Kong Li, Alexander K C Leung

**Affiliations:** 1 Department of Pediatrics, The Chinese University of Hong Kong, 6/F, Clinical Science Building, Prince of Wales Hospital, Shatin, Hong Kong, SAR, China; 2 Faculty of Medicine, The Chinese University of Hong Kong, Shatin, New Territories, Hong Kong; 3 Department of Anatomical and Cellular Pathology, The Chinese University of Hong Kong, Shatin, New Territories, Hong Kong; 4 Department of Pediatrics, The University of Calgary, 2500 University Dr NW, Calgary AB T2N 1N4, Canada

**Keywords:** Bacteria, Fungus, PICU, Pediatric intensive care, Malignancy, Mortality, Oncology, PIM2, Sepsis, Trauma, Virus, Do-not-attempt-resuscitation (DNAR), Not-responding-to-cardiopulmonary-resuscitation (NRCPR), Brain death, Organ donation

## Abstract

**Background:**

Etiologies of pediatric intensive care unit (PICU) mortality are diverse. This study aimed to investigate the pattern of PICU mortality in a regional trauma center, and explore factors associated with prolonged non-survival.

**Methods:**

Demographic data of all PICU deaths in a regional trauma center were analyzed. Factors associated with prolonged nonsurvival (length of stay) were investigated with univariate log rank and multivariate Cox-Regression forward stepwise tests.

**Results:**

There were 88 deaths (males 61%; infants 23%) over 10 years (median PICU stay = 3.5 days, interquartile range: 1 and 11 days). The mean annual mortality rate of PICU admissions was 5.8%. Septicemia with gram positive, gram negative and fungal pathogens were present in 13 (16%), 13 (16%) and 4 (5%) of these patients, respectively. Viruses were isolated in 25 patients (28%). Ninety percent of these 88 patients were ventilated, 75% required inotropes, 92% received broad spectrum antibiotic coverage, 32% received systemic corticosteroids, 56% required blood transfusion and 39% received anticonvulsants. Thirty nine patients (44%) had a DNAR (Do-Not-Attempt-Resuscitation) order with their deaths at the PICU. Comparing with non-trauma category, trauma patients had higher mortality score, no premorbid disease, suffered asystole preceding PICU admission and subsequent brain death. Oncologic conditions were the most prevalent diagnosis in the non-trauma category. There was no gunshot or asthma death in this series. Prolonged non-survival was significantly associated with DNAR, fungal infections, and mechanical ventilation but negatively associated with bacteremia.

**Conclusions:**

Death in the PICU is a heterogeneous event that involves infants and children. Resuscitation was not attempted at the time of their deaths in nearly half of the patients in honor of parents’ wishes. Parents often make DNAR decision when medical futility becomes evident. They could be reassured that DNAR did not mean “abandoning” care. Instead, DNAR patients had prolonged PICU stay and received the same level of PICU supports as patients who did not respond to cardiopulmonary resuscitation.

## Background

Improvements in resuscitation, access to advanced technologies and access to newer medications have led to improved survival of critically ill patients in intensive care units [1-4]. Nevertheless, children are occasionally inflicted with severe and life threatening diseases, necessitating supports and treatment at a pediatric intensive care unit (PICU) [5-7]. Unfortunately, a small percentage would not survive despite optimal PICU care and support [4,8].

Hong Kong, with a population of over 7 million people, has a dual public and private system for both primary and secondary health care. Although there are 10 government (Hospital Authority [HA]) and 10 private hospitals providing general pediatric inpatient service, the HA system provides the majority of inpatient pediatric care. The Prince of Wales Hospital (PWH) is a university teaching/government hospital and a tertiary trauma center situated in the New Territories in Hong Kong. PWH provides tertiary pediatric intensive care unit (PICU) service for children <12 years of age to this region with a catchment population of over 1.1 million (approximately 25% were children < 12 years of age). The incidence and pattern of mortality in young children reflects the social and demographic characteristics of that city [2,8-10]. It is generally observed that severe infections significantly contribute to childhood mortality in developing nations, whereas malignancy may be a key cause of mortality in industrialized or commercialized nations [8]. PICU mortality is diverse and broadly divided into two mutually exclusive categories: trauma and non-trauma etiologies [11,12]. This study aimed to investigate the pattern of mortality in young children admitted to a PICU in a regional trauma center, and explore factors associated with prolonged non-survival.

## Methods

The criteria for PICU admissions include cardiopulmonary insufficiency/failure, neurological deterioration, and concerns of emergency care and/or general pediatric staff. Cases were identified from the hospital’s computerized auditing system and from the PICU database of every admission to the unit. The PICU database was audited monthly. Every child who died in PICU from October 2002 (PICU opening) to December 2012 was recruited. Data were collected from patient records, hospital Clinical Management System (CMS) and autopsy reports, which included age, gender, duration in PICU stay, primary diagnosis, cause of death, mode of death, underlying conditions; use of inotropes, systemic antibiotics, anticonvulsants and systemic corticosteroids; ventilatory support; and blood product transfusions. The data also included patients’ clinical conditions such as asystole preceding PICU admission, diagnosis of encephalitis or raised intracranial pressure (ICP), disseminated intravascular coagulopathy (DIC) and multi-organ system failure (MOSF). The Paediatric index of mortality (PIM2) score was calculated for each patient (http://www.sfar.org/scores2/pim22.html)[13]. According to PIM2, high risk diagnoses include cardiac arrest preceding ICU admission, severe combined immune deficiency, leukemia or lymphoma after first induction, spontaneous cerebral hemorrhage, cardiomyopathy or myocarditis, hypoplastic left heart syndrome, HIV infection, liver failure, and neuro-degenerative disorder. Low risk diagnoses include asthma, bronchiolitis, croup, obstructive sleep apnea and diabetic ketoacidosis. Primary diagnoses were divided into 3 categories: oncologic, injury and miscellaneous.

The respiratory viruses were identified using conventional diagnostic methods, including direct immunofluorescence testing (DIFT) on respiratory samples (e.g. nasopharyngeal aspirates [NPAs]; broncho-alveolar lavages [BALs], tracheal aspirates [TAs], and oral swabs), with confirmation and typing by type-specific DIFT and viral culture. The results of all other bacterial and fungal cultures on respiratory specimens (including tracheal aspirate, pleural and sputum specimens), blood, cerebrospinal and deep wound cultures were recorded. Mode of death was classified into two categories: “Do Not Attempt Resuscitation (DNAR)” and “Not Responding to Cardiopulmonary Resuscitation (NRCPR)”. There were 5 data abstractors and inter-rater variability was controlled by standard procedures. The Ethics Committee of the Chinese University of Hong Kong approved this anonymous review with no patient identity in the manuscript, and that patient consent was not necessary for this retrospective survey. Numerical data were compared with Mann–Whitney test and categorical data with Fisher exact test. All comparisons were made two-tailed, and *p*-values less than 0.05 considered statistically significant.

## Results

From October 2002 to December 2012, 88 deaths occurred in 1505 admissions to the PICU (Table 1: males 61%; infants 23%; median PICU stay = 3.5 days, IQR: 1 and 11 days). The mean annual mortality rate of PICU admissions was 5.8% (Table 2). There was no significant change in annual mortality over the past 10 years. The median PIM2 score was 20%. Ninety percent of these patients were ventilated, 75% required inotropes, 92% received broad spectrum antibiotic coverage, 32% received systemic corticosteroids, 56% blood transfusion and 39% anticonvulsants. Thirty nine patients (44%) had a DNAR (Do-Not-Attempt-Resuscitation order) prior to their death at the PICU, but few were actively withdrawn.

**Table 1:** Clinical data of patients who died in the PICU

**Characteristic**	**Trauma (n = 9)**	**Non-trauma (n = 79)**	***P***
Median age, year (interquartile range)	5.6 (2.4, 10.7)	4.0 (0.9, 7.4)	0.20
Male, n (%)	8 (88%)	46 (58%)	0.15
PIM2 score%, median (interquartile range)	90 (60, 92)	12 (5, 56)	0.002
Oncological diagnosis, n (%)	0 (0%)	24 (30%)	0.11
Pathogens in blood culture, n (%):			
Gram + bacteria	0 (0%)	13 (16%)	0.35
Gram – bacteria	0 (0%)	13 (16%)	0.35
Fungal	0 (0%)	4 (5%)	1.00
Virus isolation, n (%)	0 (0%)	25 (32%)	0.055
Mechanical ventilation, n (%)	9 (100%)	71 (90%)	1.00
Inotropes, n (%)	8 (89%)	59 (75%)	0.68
Anticonvulsants, n (%)	3 (33%)	32 (41%)	1.00
Blood transfusion, n (%)	4 (44%)	45 (57%)	0.50
Platelets or fresh frozen plasma, n (%)	5 (56%)	49 (62%)	0.73
Disseminated intravascular coagulopathy, n (%)	3 (33%)	37 (47%)	0.50
Systemic antibiotics, n (%)	6 (67%)	75 (95%)	0.022
Systemic corticosteroids, n (%)	0 (0%)	28 (35%)	0.052
PICU survival, median (interquartile range)	2.0 (1.0, 6.0)	3.0 (1.0, 11.0)	0.84
Repeated PICU admission, n (%)	0 (0%)	19 (24%)	0.20
DNAR, n (%)	5 (56%)	34 (43%)	0.50
NRCPR, n (%)	4 (44%)	45 (57%)	
Brain death confirmed, n (%)	4 (44%)	7 (9%)	0.011
Asystole before PICU, n (%)	5 (56%)	10 (13%)	0.006
Post-mortem examination, n (%)	3 (33%)	22 (28%)	0.71

**Table 2:** PICU deaths as a percentage of the annual PICU admissions

**PICU admissions**	**Total**	**Mortality**
From Oct 2002	33	3 (9.1)
2003	122	10 (8.2)
2004	155	7 (4.5)
2005	112	4 (3.6)
2006	127	8 (6.3)
2007	144	11 (7.6)
2008	138	11 (8.0)
2009	140	4 (2.9)
2010	149	6 (4.0)
2011	198	12 (5.6)
2012	187	12 (8.3)
**Total**	**1505**	**88 (5.8)**
*p* for trend		>0.5

Twenty four were oncological diagnoses, 9 were trauma/injuries and 55 miscellaneous diagnoses which included various infections, one case each of acute disseminated encephalomyelitis, status epilepticus, dilated cardiomyopathy, hypertrophic cardiomyopathy, liver failure, post-operative bleeding, mucopolysaccharidosis type I, partial pyruvate dehydrogenase deficiency, tetrahydrobiopterin deficiency with toxic epidermolysis, spinal muscular atrophy with aspiration pneumonia, Down syndrome, multiple congenital anomalies and respiratory failure, and two cases of undetermined etiology. There was no PIM2 low-risk-diagnosis in this series (http://www.sfar.org/scores2/pim22.html)[13].

### Infections

Eighty one (92%) of patients had been treated with antibiotics for clinical infection. Septicemia with gram positive, gram negative and fungal pathogens were present in 13 (16%), 13 (16%) and 4 (5%) of these patients, respectively. The most common gram negative pathogens isolated in the blood cultures were *pseudomonas aeruginosa* (n = 5), E. coli (n = 3), *enterobacter* (n = 2), *stenotrophomonas* (n = 2) and *chryseobacterium* (n = 2). The most common gram positive pathogens isolated in blood was coagulase-negative *staphylococcus species* (CNSS) (n = 5). There were two case of pneumococcal deaths associated with multi-organ system failure and hemolytic uremic syndrome. Among the fungal blood cultures were *candida parasilosis*, *candida albicans*, *candida tropicalis*, *penicillium marneffei*; 2 also had concurrent bacterial septicemia.

Viruses were isolated in 25 patients (28%); 7 of these patients also had concurrent bacterial septicemia and 10 patients with underlying hematologic/oncologic diagnoses. The viruses included cytomegalovirus (n = 8), Ebstein-Barr virus (n = 4), herpes simplex type 1 (n = 3), human herpesvirus type 6 (n = 1), human herpesvirus type 7 (n = 1), respiratory syncytial virus (n = 3), influenza A (n = 1), parainfluenza type 3 (n = 1), adenovirus (n = 3), norovirus (n = 1), coxsackie virus (n = 1) and human metapneumovirus (n = 1). There were no deaths associated with meningitis.

### Oncology

Hematological and miscellaneous malignancies were a prevalent category associated with PICU deaths (including acute lymphoblastic leukemia, acute myeloblastic leukemia, chronic myeloblastic, lymphoma, hemophagocytic lymphohistiocytosis, Langerhans cell histiocytosis, medulloblastoma, glioblastoma, hepatoblastoma, neuroblastoma and mixed germ cell tumor. Septicemia was associated with hematological/malignant conditions. The majority of these patients did not present immediately post-bone marrow transplantation.

### Trauma

Nine deaths (10%) were due to trauma/injuries. Causes include traumatic head injury from fall (n = 4), strangulation (n = 1), suspected non accidental injury and cerebral hemorrhage (n = 1), submersion injury (n = 2) and suffocation (n = 1). There was no gunshot death or road traffic injury. Comparing with non-trauma diagnoses, trauma patients had higher PIM2 scores (p = 0.002), were more likely to present with cardiac arrest preceding ICU admission, and to have brain death subsequently (Table 1). Trauma deaths typically involved healthy boys and not involving any pathogens. There were only 2 cases of organ donation following brain deaths and both were from non-Chinese families (US and Japanese).

### Factors associated with length of PICU stay

Univariate analysis by log rank test and Kaplan-Meier plot and multivariate Cox-Regression forward stepwise test showed prolonged non-survival (length of PICU stay) was significantly associated with DNAR [18.6 versus 7.5 days, p < 0.001], fungal infections [29.6 versus 8.3 days, p = 0.002], and mechanical ventilation [13.7 versus 1 day, p < 0.001], but negatively associated with bacteremia [4.6 versus 15.5 days, p = 0.031] (Tables 3, 4 and 5).

**Table 3:** Length-of-survival analysis by univariate log rank (Mantel-Cox) test

	**Mean (standard error) days**	**95% confidence interval**	***P***
DNAR	18.6 (5.2)	8.4-28.8	<0.001
NRCPR	7.5 (3.1)	1.4-13.6	
No fungal infection	8.3 (2.3)	3.8-12.9	0.002
Fungal isolates	29.6 (10.8)	8.4-50.8	
No bacteremia	15.5 (4.0)	7.7-23.3	0.031
Bacteremia	4.6 (1.4)	2.0-7.3	
No antibiotics	1.7 (1.1)	0.0-3.8	0.008
Systemic antibiotics	13.4 (3.2)	7.2-19.7	
No ventilation	1.0 (0.4)	0.2-1.8	<0.001
Mechanical ventilation	13.7 (3.2)	7.4 (19.9)	

Length-of-survival was not associated with gender, oncological diagnosis, trauma, virus, bacteria (gram positive or gram negative), asystole preceding PICU admission, use of inotrope, anticonvulsant, seizures, raised intracranial pressure, encephalitis, disseminated intravascular coagulopathy, transfusion of blood or blood products.

One outlier with acute disseminated encephalomyelitis and respiratory failure stayed at the PICU for 1038 days. He was excluded from the calculation.

**Table 4:** Length-of-survival analysis by multivariate Cox-Regression forward stepwise test

	**B (SE)**	**Exp (B)**	**95% CI for Exp (B)**	***p***
NRCPR	0.61	1.84	1.18-2.86	0.007
Fungal isolates	−0.79	0.45	0.26-0.80	0.007
Bacteremia	0.59	1.81	1.08-3.03	0.024
Mechanical ventilation	−1.01	0.36	0.17-0.80	0.011

Exp (B): Hazard ratio for death.

**Table 5:** Multivariate Cox-Regression forward stepwise test showed none of the following factors were associated with decision for NRCPR or DNAR

Variables*	**Score**	**p**
Number of prior PICU admissions	0.912	0.34
Sex	0.001	0.98
Age	0.305	0.58
Oncologic diagnosis	0.43	0.51
Trauma	0.51	0.47
Virus	0.001	0.97
Fungus	1.80	0.18
Bacteria in BC	0.62	0.43
Gram positive bacteria in BC	1.14	0.29
Gram negative bacteria in BC	0.021	0.89
Any bacteria	0.13	0.72
Any Gram positive bacteria	1.35	0.25
Any Gram negative bacteria	0.23	0.63
Any infection	0.22	0.64
Mechanical ventilation	1.33	0.25
Asystole preceding PICU	0.88	0.35
Inotrope usage prior to CPR	0.12	0.73
Anticonvulsant usage	1.19	0.28
Seizure/encephalitis/raised intracranial pressure	1.87	0.17
Transfusion	2.01	0.16
Platelets or fresh frozen plasma transfusion	1.83	0.18
Disseminated intravascular coagulopathy	0.014	0.91
Systemic antibiotics	0.007	0.94
Systemic corticosteroid	0.074	0.79
PIM 2	0.78	0.38

*Residual Chi-Squares are not computed because of redundancies.

## Discussion

PICU admissions are generally associated with a low but definite risk of mortality [2,14-16]. There has been no significant change in trend and the low mortality rates are similar to those reported from the United Kingdom (5.1%) and Europe (5.8%) [2,4,14,16]. Infants account for one-fourth and males account for 61% of PICU deaths. The median PICU stay between admission and death was generally brief (median stay 3.5 days) and compatible with durations reported in other series [2,4,17]. Gender disparity in childhood morbidity has been demonstrated in a large study involving 92,332 pediatric admissions [18]. There is no relevant demographic difference between the two genders in terms of mortality in this series.

Patients who die in the PICU generally have multi-organ system involvement as evinced by the high rates of mechanical ventilation, inotrope and broad antibiotic usage. Septicemia is associated with a number of PICU deaths and may involve bacterial, fungal and viral pathogens [19]. Nevertheless, some patients have received antibiotics prior to PICU admissions, rendering the blood cultures negative despite the clinical presentation being consistent with sepsis. Importantly, adequate coverage for gram negative bacteria, staphylococci, streptococci, fungi and cytomegalovirus should be considered especially in immuno-compromised patients and patients with hematologic/oncologic disorders [20,21]. Sensible initial antimicrobial coverage targeting potential pathogens must be promptly instituted.

With or without infections, hematologic/oncologic diagnoses were the leading category among the non-trauma PICU deaths, and majority had hematological malignancies [20]. Our findings were consistent with data described at other PICUs [9,10,22-24]. Trauma is a common and important cause of pediatric mortality [2,7,11,12,25]. The mechanisms of injury vary widely [8]. Falls account for nearly half of the deaths. Drowning or submersion injury represents another small but potentially preventable cause of mortality [6,7,11]. In this series, no infants died from trauma or non-accidental injury. Interestingly, there has been no pediatric gunshot death in Hong Kong due to strict local gun-control regulations. This is very different from countries where gunshots contribute to a significant part of pediatric death [7,8,11,26] PICU deaths associated with trauma showed a high percentage of male due possibly to curiosity and accident-prone behavior in boys [27]. More than half of the trauma deaths in our study were due to head injuries and subsequent brain-death. In addition, PIM2 score of the trauma associated deaths was significantly higher than that of the non-trauma related deaths. This might be due to the fact that significantly more patients in the trauma group presented in asystole before PICU admission, which contributes to “high risk diagnosis” in calculation of the PIM 2 score [13].

Deaths in PICU patients with metabolic diseases were relatively rare and often difficult to confirm. A similar study in the UK showed that 4.4% of the PICU non-survivors were diagnosed of metabolic diseases [2]. Chromosomal abnormalities and neurodegenerative diseases are also uncommon in our group of patients.

The decision and implementation of an end-of-life plan is a multi-faceted process that takes time and patience to eventuate [14,16,28]. Resuscitation was not attempted at the time of their deaths in nearly half of the patients in honor of caregivers’ wishes. Parents often make timely DNAR decision when futility of treatment becomes evident. As a result, DNAR patients may paradoxically appear to take a lot longer to inevitably die. Despite prolonged non-survival, caregivers can be assured that DNAR patients generally received the same level of PICU supports including hemodynamic monitoring, mechanical ventilation, inotrope and antimicrobial usage as NRCPR patients during their PICU course.

There were only two PICU cases of organ donation following brain deaths, and both patients were from non-Chinese families (US and Japanese). Organ donations are rare in Chinese families possibly related to ethnic beliefs [29]. These patients typically did not have active infection.

Limitation of this study was the relative small sample size and heterogeneity despite the long study period of over 10 years. This may be due to the advanced medical care and relative small pediatric population in the aging Hong Kong demographics. In addition, difficulties were encountered in retrieving some of the patients’ records due to the retrospective nature of the study. Nevertheless, data from CMS and autopsy reports are highly reliable. Lastly, our study only investigates PICU deaths and excludes children who die prior to hospitalization or PICU admission.

## Conclusion

In conclusion, PICU admissions were associated with a 5.8% mortality rate. Death in the PICU is a heterogeneous event that involves infants and children. Resuscitation was not attempted at the time of their deaths in nearly half of the patients in honor of parents’ wishes. Parents often make DNAR decision when medical futility becomes evident. Despite prolonged non-survival, DNAR patients generally received the same level of PICU supports as NRCPR patients during their PICU course.

## Competing interests

The authors declare that they have no competing interests.

## Authors’ contributions

KLHH is the principal author. TCWP is the scientific staff who performed detailed statistical analysis. WW, KLC, HMC, TFL, CKL wereinvolved in the care of these patients and contributed to the design and drafting of this manuscript. KFT is the pathologist involved in post-mortum analysis of some of these patients. KKL, HWM, KWT, WKW, HFW wereinvolved in the data collection and analysis. AKCL was involved in the interpretation of the data and revision of the manuscript critically for important intellectual content. All authors read and approved the final manuscript.

## Pre-publication history

The pre-publication history for this paper can be accessed here:

http://www.biomedcentral.com/1471-2253/13/43/prepub
